# Effect of dietary supplementation of garlic, ginger and their combination on feed intake, growth performance and economics in commercial broilers

**DOI:** 10.14202/vetworld.2016.245-250

**Published:** 2016-03-08

**Authors:** V. K. Karangiya, H. H. Savsani, Shrikant Soma Patil, D. D. Garg, K. S. Murthy, N. K. Ribadiya, S. J. Vekariya

**Affiliations:** 1 Department of Animal Nutrition, College of Veterinary Science and Animal House, Jungadh Agricultural University, Junagadh, Gujarat, India; 2 Department of Animal Husbandry and Extension Education, College of Veterinary Science and Animal House, Jungadh Agricultural University, Junagadh, Gujarat, India

**Keywords:** body weight, broiler chickens, feed intake, garlic, ginger

## Abstract

**Aim:**

The present study was carried out to evaluate the effect of supplementation of garlic, ginger and their combination in the diets of broiler chickens and assessment in terms of feed intake, growth performance and economics of feeding.

**Materials and Methods:**

A total of 240 1-day-old Cobb-400 broiler chicks were randomly assigned to four dietary treatments each with three replicates of 20 chicks per replicate (n=60). Four experimental diets were formulated in such a way that control diet (T_1_) contained neither ginger nor garlic. While, birds in group T_2_ and T_3_ were fed with diets containing 1% garlic and ginger, respectively. Diet 4 (T_4_ group) contained a combination of 1% of garlic and ginger. The feeding experiment was carried out for 42 days, and different parameters evaluated includes feed intake, weight gain, feed conversion ratio, gut morphometry, and economics of feeding in terms of return over feed cost (ROFC) and European Performance Efficiency Index.

**Results:**

Feed intake of experimental birds in ginger and mixture of garlic and ginger supplemented groups, i.e., T_3_ and T_4_ groups have significantly (p<0.05) higher feed intake as compared to control. While, feeding of garlic have non-significant effect on feed intake as compared to other groups. A body weight gain (g/bird) was found to be significantly (p<0.05) higher in garlic (T_2_ group) and ginger (T_3_ group) supplemented group as compare to control and garlic and ginger mixture supplemented group (T_4_ group). Feed conversion ratio was significantly (p<0.05) lower in ginger (T_3_ group) supplemented group as compare to other groups. Mean villi length, villi width and cryptal depth were significantly (p<0.05) higher in T_3_ group than rest of all three groups, indicating increased absorptive surface area. ROFC was significantly (p<0.05) lower in T_3_ and T_4_ groups as compare to control. However, it was not significantly different between control and T_2_ group.

**Conclusion:**

On the basis of the results of the study, it is concluded that supplementation of garlic improves the performance of broilers when added at the rate of 1% of broiler ration and can be a viable alternative to antibiotic growth promoter in the feeding of broiler chicken.

## Introduction

Feed is the major component of total costs of poultry venture as 80% of the total expenditure is on procurement of feed [1]. Feed additives are a group of nutrient and non-nutrient compounds which helps in improving the efficiency of feed utilization and thus reducing the high cost of feed. In the past, antibiotics were the most routinely used feed additives. However, nowadays use of antibiotics is not only limited but their use in livestock and poultry industry also have been banned in many countries due to the reasons like alteration of natural gut microbiota and drug resistance in bacteria and humans. As a result, to replace them without adversely affecting the performance of birds, natural growth promoters such as prebiotics, probiotics, synbiotics, enzymes, plant extracts, etc., can be used to feed the broilers [2].

Garlic and ginger as natural growth promoters can be potential alternatives for common artificial growth promoters like antibiotics [3]. Ginger is the rhizome of the plant *Zingiber officinale*, consumed as a delicacy, medicine, or spice. Preliminary research indicates that nine compounds found in ginger may bind to serotonin receptors which may influence gastrointestinal function. Research conducted *in-vitro* shows that ginger extract might control the quantity of free radicals and the peroxidation of lipids [4] and have anti-diabetic properties [5]. Garlic (*Allium sativum*) has been used as a spice and a native medicine for many years. It has possessed antibacterial, antifungal, antiparasitic, antiviral, antioxidant, anticholesteremic, anti-cancerous, and vasodilator characteristics [6]. Ginger and garlic supplements in broiler chicken diets have been recognized for their strong stimulating effect on the immune and digestive systems in birds [7]. Recent research works on ginger and garlic formulations as feed additives have shown encouraging results in regards to weight gain, feed efficiency, lowered mortality and increased livability in poultry birds [8,9].

Different workers have tried at different levels of garlic and ginger in the diet of birds and but most consistent results were obtained at about 1% level [10-12] and supplementation of these products beyond 1% of ration may also have a negative effect on overall cost of feeding. As a result, they are incorporated at the level of 1% in the diet of broilers. On the other hand, studies on their use as mixtures in the diet of birds have produced inconsistent results. Therefore, this study was planned to generate more information about the effect of using garlic and ginger alone and in combination on performance and economics of supplementation in the diet broiler chickens.

## Materials and Methods

### Ethical approval

This research was carried out as a part of M.V.Sc. research after the approval of competent authority of the Director of research and Dean P.G. Studies, Junagadh Agricultural University, Junagadh.

### Location of study

The experiment was carried out at the at modern poultry farm located at Mangrol, city of Junagadh district which lies approximately on latitude 70.12’E and longitude 21.12’N with an average elevation of 18 m (59 feet) above sea level. This region of South Saurashtra sub-zone comes under Zone-XIII of agro climatic zone of India, which includes only Junagadh district. A climate is dry sub-humid, and adverse climatic conditions prevail in this region with minimum and maximum temperature ranging from 28°C to 38°C in summer and 10°C to 25°C in winter months [13]. The average annual rainfall ranges from 1000 to 1200 mm.

### Source and processing of ginger and garlic

Groundnut ginger and garlic used in this study was bought from a local market in raw form. Then, it was cut into smaller pieces and dried sufficiently in the sunlight. After drying, required amount of ginger and garlic was prepared by fine grinding and passing through 1 mm sieve.

### Experimental birds and diets

About 1-day-old 240 broiler chicks of cobb-400 strain with average body weight 40.00-40.28 g were wing banded and distributed randomly into four groups having three replicates of 20 birds each by randomized block design and allocated to four dietary treatments as T_1_, T_2_, T_3,_ and T_4_. Experimental birds in group T_1_ were fed with conventional concentrate mixture while birds in T_2_, T_3,_ and T_4_ groups were fed on concentrate mixture supplemented with 1% garlic, 1% ginger and mixture of 1% garlic and 1% ginger, respectively. Ingredient compositions of these starter and finisher rations are presented in Tables-1 and 2, respectively.

**Table-1:** Ingredient and chemical composition of broiler starter feeds.

Feed ingredient (kg)	T_1_	T_2_	T_3_	T_4_
Maize	63	62	62	61
Soybean meal	29	29	29	28
Meat and bone meal	3	3	3	3
Vegetable oil	2.5	2.5	2.5	2.5
Lime stone	1.2	1.2	1.2	1.2
Dicalcium phosphate	0.8	0.8	0.8	0.8
Garlic	0	1	0	1
Ginger	0	0	1	1
Salt	0.45	0.45	0.45	0.45
Methionine	0.27	0.27	0.27	0.27
Lysine	0.20	0.20	0.20	0.20
Vitamin	0.05	0.05	0.05	0.05
Mineral mixture	0.1	0.1	0.1	0.1
Calcium carbonate	0.15	0.15	0.15	0.15
Liver tonic	0.05	0.05	0.05	0.05
Total	100.77	100.77	100.77	100.77
Chemical composition (% DM basis)				
DM	92.45	92.44	92.66	93.34
CP	23.18	23.30	23.12	23.24
EE	3.36	3.52	3.40	3.56
CF	4.36	4.61	4.55	4.20
NFE	61.3	60.96	60.96	60.87
TA	7.80	7.61	7.97	8.13
Ca	1.11	1.09	1.09	1.10
P	0.86	0.91	0.90	0.89
ME (Kcal)	3176	3176	3176	3176

Tracemin CB: Each 1 kg contain: Manganese - 90 g, Zinc - 80 g, Iron - 90 g, Copper - 15 g, Iodine - 2 g and Selenium - 300 mg, Vitamin (Groviplex powder): Each 100 g contains: Vitamin B2 - 1.25 g, Vitamin B6 - 0.620 g, Vitamin B12 - 6.25 g, Nicotinamide - 18.75 g, Lysine monohydrochloride - 10.00 g, Choline bitartarate - 15.82 g, Calcium pantothenate - 1.51 g. DM=Dry matter, OM=Organic matter, CP=Crude protein, CF=Crude fiber, NFE=Nitrogen free extract, ME=Metabolic energy

**Table-2:** Ingredient and chemical composition of broiler finisher feeds.

Feed ingredient (kg)	T_1_	T_2_	T_3_	T_4_
Maize	65	64	64	64
Soybean meal	25	25	25	24
Meat and bone meal	3	3	3	3
Vegetable oil	4	4	4	4
Lime stone	1.1	1.1	1.1	1.1
Dicalcium phosphate	0.8	0.8	0.8	0.8
Garlic	0	1	0	1
Ginger	0	0	1	1
Salt	0.45	0.45	0.45	0.45
Methionine	0.25	0.25	0.25	0.25
Lysine	0.15	0.15	0.15	0.15
Vitamin	0.05	0.05	0.05	0.05
Mineral mixture	0.1	0.1	0.1	0.1
Calcium carbonate	0.15	0.15	0.15	0.15
Liver tonic	0.05	0.05	0.05	0.05
Total	100.10	100.10	100.10	100.10
Chemical composition (% DM basis)				
DM	92.22	92.25	92.24	92.51
CP	20.15	20.27	20.19	20.28
EE	3.27	3.40	3.36	3.47
CF	4.43	4.45	4.53	4.74
NFE	62.84	62.79	63.02	62.94
TA	9.31	9.09	8.90	8.57
Ca	1.16	1.14	1.17	1.14
P	0.97	0.96	0.99	0.90
ME (Kcal)	3281	3281	3281	3281

Tracemin CB: Each 1 kg contain: Manganese - 90 g, Zinc - 80 g, Iron - 90 g, Copper - 15 g, Iodine - 2 g and Selenium - 300 mg, Vitamin (Groviplex powder): Each 100 g contains: Vitamin B2 - 1.25 g, Vitamin B6 - 0.620 g, Vitamin B12 - 6.25 g, Nicotinamide - 18.75 g, Lysine monohydrochloride - 10.00 g, Choline bitartarate - 15.82 g, Calcium pantothenate - 1.51 g. DM=Dry matter, OM=Organic matter, CP=Crude protein, CF=Crude fiber, NFE=Nitrogen free extract, ME=Metabolic energy

### Feeding and management procedures

All the experimental birds were reared in well ventilated shed in deep litter pens and kept under uniform management conditions. The reference and test concentrate mixtures and clean drinking water were supplied to the birds *ad libitum* throughout the study period to meet the nutrient as per BIS [14]. All the birds were weighed weekly in the morning, before feeding and watering. Feed intake was calculated by measuring the amount of feed offered and residue left after 24 h. Feed conversion ratio was calculated by dividing the feed intake by weight gain. Vaccination and other routine poultry management practices were carried out neatly.

### Gut morphometry

At the end of the experiment, two birds per replicate were randomly selected and slaughtered by decapitation to study gut morphometry. Sections of the jejunum were harvested and fixed in 10% formalin. Histological sections were cut and stained with hematoxylin and eosin stain. From each section, two randomly selected villi were measured in each slide per microscopic field. Two fields were used for measurement of villi lengths, widths and cryptal depths under the microscope using graduated eyepiece with a (×10) objective lens [15]. The villus height was measured from the tip of the villus to the villus-crypt junction. The villus width was defined as the distance from the outside epithelial edge to the outside of the opposite epithelial edge along a line passing through the vertical midpoint of the villus. The crypt depth was defined as the depth of the invagination between adjacent villi [16].

### Return over feed cost (ROFC)

It is calculated by subtracting the total feed cost from the income from the sold bird on live weight basis during from rearing period of 42-day. While, European Performance Efficiency Index (EPEI) was calculated by following formula [17]:


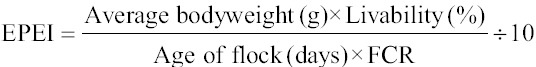


### Chemical and statistical analysis

Samples of feeds were milled to pass through a 1 mm sieve and then analyzed following the methods of AOAC [18] to determine dry matter by the oven drying method (934.01), organic matter by muffle furnace incineration (967.05), crude protein by Kjeldahl method (984.13) (N × 6.25), ether extract (920.39), ash (942.05). The data collected on various parameters were analyzed using the method of Snedecor and Cochran [19].

## Results and Discussion

### Nutrient composition of experimental rations

Nutrient composition of different concentrate mixtures is given in Tables-1 and 2. All the feeds were found to be isonitrogenous and comparable with respect to their proximate composition.

### Feed intake (g/bird)

Average total feed intake of experimental birds in T_3_ and T_4_ groups was significantly (p<0.05) higher as compared to T_1_ and garlic supplemented T_2_ group (Table-3). However, feed intake in T_1_ and T_2_ was similar and did not differ significantly. It has shown that there is no adverse effect of smell and/or taste of garlic and ginger on the palatability of feed in the diets of broilers.

**Table-3:** Proximate composition of garlic and ginger (on % DMB).

Particulars	Garlic	Ginger
DM	85.25	87.39
OM	74.64	76.69
CP	7.41	5.85
CF	1.83	2.93
EE	2.41	1.35
NFE	85.81	87.58
Total ash	2.54	2.29

EE=Ether extract, DM=Dry matter, OM=Organic matter, CP=Crude protein, CF=Crude fiber, NFE=Nitrogen free extract, DBM=Diamondback moth

The results are in agreement with different workers who has that reported that ginger powder in the broiler’s diets had a significant positive effect on feed consumption [20,21]. Similarly, different workers have reported non-significant effect of garlic supplementation on feed intake in broilers [22-24].

### Body weight gain (g) and feed conversion ratio

Total body weight gain (g) of experimental birds supplemented with garlic (T_2_) and ginger (T_3_) showed significantly (p<0.05) higher values as compared to control and combination of garlic and ginger (T_4_) supplemented group (Table-3). Whereas, non-significant difference was observed between T_1_ and T_4_ groups. The improvement in weight gain of experimental birds fed with garlic powder may be due to the action of compounds like allicin and oregano sulfur compound responsible for inhibition of pathogenic bacteria and fungi results into the improved gut environment [25]. Improvement in body weight gain of broiler chicks fed on ginger might be due to the active components present in the ginger which stimulates digestive enzymes and improves overall digestion and thus leads to increased body weight gain. It has been established fact that ginger in the diets stimulate lactic acid bacteria and decreases pathogenic bacteria such as mesophilic aerobic, coliform and *Escherichia coli* and thus improves absorption of nutrients leads to better weight gain of the birds [26]. The results are consistent with those of Mohamed *et al.*, Sadeghi *et al.*, Arshad *et al*. [21,27,28] who stated that use of ginger to the diet of broilers had a significant (p<0.05) positive effect on the body weight gain as compared to the control.

Experimental birds in T_4_ group showed significantly (p<0.05) higher average FCR as compare to T_1_, T_2_ and T_3_ groups (Table-3). The supplementation of garlic and ginger fed alone in the diet of birds does not exert any significant effect on FCR as compared to control. Although, better FCR was observed in T_3_ group followed by T_2_ and T_1_ groups. These results are in accordance with the findings of Aji *et al.*, Mansoub and Nezhady [23,29] who has reported non-significant effect of garlic on FCR. These results are also in agreement with Ademola *et al.*, Thayalini *et al*. [30,31] who did not observe any significant improvements in the feed conversion ratio of broilers fed on a diet containing ginger powder as compared to the control group.

### Gut morphometry

The statistical analysis of the morphometry of the jejunum showed that values of mean villi length and width were significantly (p<0.05) higher in T_3_ group (Table-4). This represents an increase in the absorptive surface area of the intestine and thus an increased absorptive capacity resulting higher body weight gain and lower FCR in ginger supplemented group. Same was true for cryptal depth in treatment group T_3_, and it was significantly (p<0.05) higher compare to other groups. Since crypt cells are responsible for secretion of electrolytes which enhance water secretion into the intestinal lumen for the purpose of digestion, it could be inferred that digestibility of nutrient was enhanced in T_3_ group.

**Table-4:** Performance of experimental birds.

Particulars	Treatments				SEM	p value

	T_1_	T_2_	T_3_	T_4_		
Initial body weight (g)	40.10	40.00	40.02	40.12	0.04	NS
Final body weight (g)	1646.68^c^	1689.32^b^	1758.89^a^	1638.26^c^	13.63	p<0.05
Average final body weight gain (g)	1606.7^c^	1649.17^b^	1724.17^a^	1598.36^c^	28.76	p<0.05
Average weekly weight gain (g)	267.76	274.88	286.20	266.34	7.7	NS
Average total feed intake	3270.56^b^	3259.12^b^	3367.77^a^	3395.61^a^	34.28	p<0.05
Feed conversion ratio	1.90^b^	1.87^b^	1.84^b^	1.99^a^	0.03	p<0.05

Means within row with different superscript differ significantly (p<0.05). NS=Non-significant, SEM=Standard error of mean

Stem cells at the base of crypts are known to be the source of all the cells which lines the crypt and the villi. Thus, a higher cryptal depth observed in T_3_ is an indication of a higher mucosal proliferation activity for more efficient digestibility and absorption of ingested feed in this group. This study shows that ginger has ability to increase the digestive and absorptive capacity of the small intestine of commercial broilers by increasing the cryptal depth as well as the absorptive surface area of the intestine i.e.. villi length and width. These finding are in agreement with those of Oladele *et al*. [32] who stated that an increase in the absorptive capacity of intestine might be due to increase absorptive surface area which results in higher body weight gain and lower FCR of broiler birds.

### ROFC

Average ROFC in terms of (Rs./bird), income from selling of birds and feed cost of broilers under different treatment groups has been shown in Table-5. The cost of feed per kg for different treatment groups in respect to starter and finisher phases is shown in Table-6. The income from selling of the birds was significantly (p<0.05) higher in T_3_ group than the control group, but there was a non-significant difference between other supplemented groups. Feed cost during whole experimental period was significantly (p<0.05) higher in T_3_ and T_4_ group than control. While, it was non-significant for control and T_2_ group. ROFC was significantly (p<0.05) lower in T_3_ and T_4_ groups as compare to control. However, it was not significantly different between control and T_2_ group. ROFC (Rs./kg live weight) was highest in T_1_ (14.21%) then T_2_ (13.30%) followed by T3 (8.72%) and T_4_ (1.09%) suggesting comparable returns of 1% garlic with control. However, due to the higher cost of ginger, rate over feed cost is diminishing in other two groups. It indicated that ginger did not show any negative or positive effect in ROFC. The present study was in accordance with Mohammed and Yusuf [33] who has found no differences in cost of feed per kg gain for broilers on dietary supplementation of ginger.

**Table-5:** Gut morphometry of experimental birds.

Particulars	Treatments				p value

	T_1_	T_2_	T_3_	T_4_	
Villi length	25.6±2.0^c^	27.8±2.9^b^	39.3±2.3^a^	27.4±1.8^b^	p<0.05
Villi width	6.02±1.1^b^	5.76±1.1^b^	7.64±1.6^a^	7.60±1.5^a^	p<0.05
Cryptal depth	11.1±1.1^c^	21.3±2.1^a^	23.0±1.6^a^	13.7±4.3^b^	p<0.05
Villus height: Cryptal depth	2.30	1.31	1.71	2.0	

All regions, absolute units, lengths ×10 μm. Means within row with different superscript differ significantly (p<0.05)

**Table-6:** The ROFC (Rs./bird) realized under different feed supplement groups.

Particulars	T_1_	T_2_	T_3_	T_4_	p-value
Income from bird sold (Rs./bird)	107.25±2.25^b^	109.84±2.37^b^	114.39±2.05^a^	106.60±1.82^b^	p<0.05
Feed cost (Rs./bird)	83.80±1.44^b^	87.36±1.28^b^	99.03±0.88^a^	104.8±1.16^a^	p<0.05
ROFC (Rs./bird)	23.45±1.23^a^	22.48±1.18^a^	15.36±0.82^b^	1.80±0.08^c^	p<0.05
ROFC (Rs./kg live weight)	14.21	13.30	8.72	1.09	

*Means within row with different superscript differ significantly (p<0.05). ROFC=Return over feed cost

### EPEI

The EPEI for performance of the broilers is given in Tables-7 and 8. The EPEI value was higher in T_3_ (223.94) which was followed by T_2_ (211.58), T_4_ (196.22) and T_1_ (192.98). EPEI value was significantly (p<0.05) higher in T_3_ and T_2_ groups than the control group. While, there was a non-significant difference between T_1_ and T_2_ group. The higher EPEI value corresponds to higher average body weight; superior livability and higher feed conversion ratio in a stipulated period of experimental trial and thus indicates overall economics feeding in birds [34]. Hence, on the basis of EPEI, it can be stated that supplementation of ginger in the diet of birds was found to be more economical than other groups.

**Table-7:** Cost of feeds under different feed supplement groups.

Type of feed	T_1_	T_2_	T_3_	T_4_
Broiler starter (Rs./kg)	25.56	27.06	29.56	31.06
Broiler finisher (Rs./kg)	25.22	26.72	29.22	30.72

**Table-8:** EPEI of broilers fed with garlic, ginger and their combination under different treatment.

Particulars	Treatments				p value

	T_1_	T_2_	T_3_	T_4_	
EPEI	192.98±4.28^c^	211.58±5.36^b^	223.94±6.22^a^	196.22±4.92^c^	p<0.05

Means within row with different superscript differ significantly (p<0.05). EPEI=European performance efficiency index

## Conclusion

At the end of the experiment, on the basis of the performance of broilers in respect to feed intake, body weight gain, FCR, gut morphometry, it is observed that garlic supplementation was superior in comparison to ginger and mixture of garlic and ginger. Although ROFC was lower in ginger supplement group, it has significantly higher EPFI value as compared to other groups. Therefore, it is concluded that supplementation of garlic improves performance of broilers when added at the rate of 1% of broiler ration and can be a viable alternative to antibiotic growth promoter in the feeding of broiler chicken.

## Authors’ Contributions

HHS has conceptualized the aim of the study, designed and supervised the experiment. VKK executed the experiments and conducted statistical analyses. SSP, DDG and KSM has drafted, corrected and revised the manuscript. NKR and SJV have carried out laboratory analysis of feed samples. All authors read and approved indicating that they are collectively derived the final manuscript.
